# Centralized healthcare database for ensuring better healthcare: Are we lagging behind?

**DOI:** 10.12669/pjms.40.3.9084

**Published:** 2024

**Authors:** Sundus Tariq, Saba Tariq, Ahmad Adnan Shoukat

**Affiliations:** 1 Sundus Tariq, Department of Physiology, International School of Medicine, Istanbul Medipol University, Research Institute for Health Sciences and Technologies (SABITA), Istanbul, Turkey; 2 Saba Tariq, Department of Pharmacology and Therapeutics, University Medical & Dental College, The University of Faisalabad, Faisalabad, Pakistan, University of Birmingham, Birmingham, United Kingdom; 3 Ahmad Adnan Shoukat, Department of Biomedical Engineering and Bioinformatics, School of Engineering and Natural Sciences, Istanbul Medipol University, Istanbul, Turkey

Database is an organized set of large information gathered and stored electronically to be retrieved whenever required. The systematically stored information can be accessed, analyzed, updated or moved to other databases as per the requirement. A centralized healthcare database (CHD) is a memory house of health data from a wider population, where information such as health records, financial data, billing and claims information and inventory use is not only stored systematically but can also be retrieved, analyzed, and integrated. The data can be assessed by the health care providers, researchers, policy makers, institutions and by the patients themselves.

In the United States almost 100% hospitals shifted to centralised electronic health record systems as a result of the 2009’s Health Information Technology for Economic and Clinical Health (HITECH) Act.^1^ The system was further strengthened by the Trusted Exchange Framework and Common Agreement^SM^ (TEFCA^SM^) that is responsible for establishing universal governance, policy, and technical floor for nationwide interoperability, help consumers easily and securely access their electronic health information, secure exchange of information between organizations for a better patient care, foster healthcare value and direct towards augmenting population welfare.^2^

Many countries were using Electronic Medical Record systems (EMRs), but it was only after COVID-19 that the researchers and clinicians found that the EMRs have not lived up to their full potential due to decentralization of data. This was overcome by the development of CHD.^3^

## How is it beneficial?

Development of a CHD is an efficient and quick way to search through large volumes of data composed of centralized patient information offering a complete view of a patient’s medical history, treatments, prescriptions, diagnostic test results, billing information and many more. Apart from sparing a large space in the hospital’s record rooms, it minimize cluttering. It allows interdepartmental integration, where health care providers can share patient’s information to improve coordination and treatment plans to provide best patient care. Continuous patient care can be provided even after discharge of the patient. It largely supports the telemedicine services too.

A review conducted between the years 2010 to 2019 to access the evidence based value of the electronic medical record for hospital care showed that the EMRs are efficient in reducing the costs and improving the health care quality.^4^

Maintaining a CHD reduces duplication of tests and procedures, saving time and resources while minimizing the risk of redundant treatments. Researchers can get great benefit from this CHD by designing studies after anonymizing the data and using for research purposes, aiding in understanding disease patterns, treatment outcomes, and public health trends.

CHD can be analyzed to identify trends and best practices, supporting evidence-based decision-making for healthcare policies and procedures. If employed in the form of Big Data, it can calibrate and adjust the screening and preventive protocols to reduce the burden of many diseases like cancers and metastasis.^5^

## The complexity of getting databases in our systems:

In order to get a CHD, quintillions of bytes have to be created and managed. In addition, accurate and efficient data management systems need to be linked to the CHD in order to ensure safe and efficient transfer of sensitive and confidential healthcare data. If the data is not processed with care, it results in medical errors. There is a need to employ standardized data management systems to make the system efficient and error free.^6^

The system should work with physician’s satisfaction, balancing the benefits of improving patient outcomes with the potential pitfalls of increasing physician’s burnout due to poor implementation leading to added complexity.^5^

Strong and strict security measures are required to protect patient data in CHD from the unauthorized access and breach in patient confidentiality should be prevented. There should be strict privacy regulations for CHD as is used by Trusted Exchange Framework and Common Agreement^SM^ (TEFCA^SM^).

Multicenter comparison of the large data sets require uniform health indicators. Many countries lack this and the availability of patient related information differ strongly by countries. This can be avoided by shifting from institution centered to patient centered information processing. A survey conducted across 14 countries in the year 2017 on the availability of cross-institutional e-health indicators related to patient data for healthcare professionals, patients and their caregivers showed that this information varies strongly by country. Only four countries, Finland, South Korea, Japan and Sweden showed best outcome related to availability of uniform e-health indicators.^7^

## Where do we stand?

Turkey has significantly improved its health care system under the Health Transformation Program launched by the ministry of health in 2003. The major component of this was to achieve e-health. They created a centralized health care system “saglik.net” whose objectives were to ensure standardization of healthcare data, creating and maintaining the health record of their population, data analysis, expedite the flow of information and saving resources ultimately increasing overall efficiency of the healthcare system. One of the component of this program is family medicine information system that integrated all public hospitals. They are maintaining their central database through the National Health Information System (NHIS). Ministry of Health Turkiye in collaboration with European countries is working on the Smart Open Services for European Patients (epSOS) to provide interoperability platform for sharing healthcare data. The consortium of the epSOS project composes of 48 partners form 23 European countries.^8^

India is working efficiently to maintain CHD. A recent example is an online centralized database management system for a newborn sickle cell program (NBSP), which is dedicated to a highly prevalent disease in India. More than 20 million sickle cell affected individuals reside in the country. This database is expected to help in eradication of sickle cell disease from India by the year 2047.^9^

Healthcare system in Pakistan is facing many challenges. The existence of two parallel systems, public and private demands balance. Where the private sector is flourishing and have successfully established their own healthcare databases, the government sector is still struggling to maintain even the basic health facilities. Stakeholders need to invest in developing the healthcare infrastructure otherwise Pakistan will continue fighting for its survival rather than improving and competing with the healthcare systems of other nations in the region.^10^

## CONCLUSION

In conclusion, while CHD offers substantial benefits in enhancing patient care, improving healthcare systems, and aiding research, the successful implementation of these databases necessitates addressing challenges related to data management, security, uniformity, and ensuring physician satisfaction to harness their full potential. Efforts to streamline and standardize these systems on a global scale can significantly improve healthcare outcomes worldwide.

## References

[1] DeSalvo K, Washington V (2016). By the numbers:Our progress in digitizing health care. Health Affairs Forefront.

[2] Official Website of the Office of the National Coordinator for Health Information Technology (ONC) [Online] 2023.

[3] Pesheva E Portrait of a virus. Harvard Medical School, News and Research. [Online] 2020.

[4] Uslu A, Stausberg J (2021). Value of the electronic medical record for hospital care:update from the literature. J Med Internet Res.

[5] Agrawal R, Prabakaran S (2020). Big data in digital healthcare:lessons learnt and recommendations for general practice. Heredity.

[6] Adane K, Gizachew M, Kendie S (2019). The role of medical data in efficient patient care delivery:a review. Risk Manag Healthc Policy.

[7] Ammenwerth E, Duftschmid G, Al-Hamdan Z, Bawadi H, Cheung NT, Cho KH (2020). International comparison of six basic eHealth indicators across 14 countries:an eHealth benchmarking study. Methods Inf Med.

[8] Turkish Health Transformation Program and Beyond (2023). [Online].

[9] Pandey A, Borah S, Chaudhary B, Rana S, Singh H, Nadkarni A (2023). NBSP:an online centralized database management system for a newborn sickle cell program in India. Front Digit Health.

[10] Muhammad Q, Eiman H, Fazal F, Ibrahim M, Gondal MF (2023). Healthcare in Pakistan:Navigating Challenges and Building a Brighter Future. Cureus.

